# Performance analysis of seven Convolutional Neural Networks (CNNs) with transfer learning for Invasive Ductal Carcinoma (IDC) grading in breast histopathological images

**DOI:** 10.1038/s41598-022-21848-3

**Published:** 2022-11-10

**Authors:** Wingates Voon, Yan Chai Hum, Yee Kai Tee, Wun-She Yap, Maheza Irna Mohamad Salim, Tian Swee Tan, Hamam Mokayed, Khin Wee Lai

**Affiliations:** 1 Department of Mechatronics and Biomedical Engineering, Lee Kong Chian Faculty of Engineering and Science, Universiti Tunku Abdul Rahman, Sungai Long, Malaysia; 2 Department of Electrical and Electronic Engineering, Lee Kong Chian Faculty of Engineering and Science, Universiti Tunku Abdul Rahman, Sungai Long, Malaysia; 3 Diagnostic Research Group, School of Biomedical Engineering and Health Sciences, School of Biomedical Engineering and Health Sciences, Faculty of Engineering, Universiti Teknologi Malaysia, 81300 Skudai, Johor Malaysia; 4 BioInspired Device and Tissue Engineering Research Group, School of Biomedical Engineering and Health Sciences, Faculty of Engineering, Universiti Teknologi Malaysia, 81300 Skudai, Johor Malaysia; 5 Department of Computer Science, Electrical and Space Engineering, Lulea University of Technology, Luleå, Sweden; 6 Department of Biomedical Engineering, Universiti Malaya, 50603 Kuala Lumpur, Malaysia

**Keywords:** Image processing, Machine learning, Cancer imaging, Biomedical engineering

## Abstract

Computer-aided Invasive Ductal Carcinoma (IDC) grading classification systems based on deep learning have shown that deep learning may achieve reliable accuracy in IDC grade classification using histopathology images. However, there is a dearth of comprehensive performance comparisons of Convolutional Neural Network (CNN) designs on IDC in the literature. As such, we would like to conduct a comparison analysis of the performance of seven selected CNN models: EfficientNetB0, EfficientNetV2B0, EfficientNetV2B0-21k, ResNetV1-50, ResNetV2-50, MobileNetV1, and MobileNetV2 with transfer learning. To implement each pre-trained CNN architecture, we deployed the corresponded feature vector available from the TensorFlowHub, integrating it with dropout and dense layers to form a complete CNN model. Our findings indicated that the EfficientNetV2B0-21k (0.72B Floating-Point Operations and 7.1 M parameters) outperformed other CNN models in the IDC grading task. Nevertheless, we discovered that practically all selected CNN models perform well in the IDC grading task, with an average balanced accuracy of 0.936 ± 0.0189 on the cross-validation set and 0.9308 ± 0.0211on the test set.

## Introduction

Worldwide, there were an estimated 19.3 million new cancer cases and almost 10.0 million cancer deaths in 2020. For women, breast cancer is now the most common type of cancer, with an estimated 2.3 million new cases each year^1^. Breast cancer is a category of disorders in which the cells of the breast multiply uncontrolled, resulting in the formation of a lump in a specific location of the breast^2^. IDC is the most common type of breast cancer, accounting for more than 80% of all cases^3^. Early detection and screening are critical for effectively preventing breast cancer. Breast cancer screening consists of three procedures: mammography, breast magnetic resonance imaging (MRI), and breast ultrasonography^4^. If suspicious tissue is detected, physicians extract it via biopsy for further histologic examination. After tissue extraction, three steps are performed prior to histological grading: (1) formalin fixation, (2) paraffin section embedment, and (3) haematoxylin and eosin staining^5^.

The primary three prognostic markers that determine a breast cancer treatment are (1) lymph node (LN) status, (2) tumour size and (3) histological grade^6^. Multiple studies have shown that the prognosis indicated by the histological grade is equal to the lymph node (LN) condition but higher than the tumour size^7,8^. It is established that the prediction accuracy for clinical outcomes improved when both histological grade and LN condition are applied together^9^. Frkovic-Grazio and Bracko^10^ found that the histology grade predicted tumour behaviour accurately, especially for early small tumours. Schwartz et al.^11^ revealed that high-grade breast cancer patients who underwent mastectomy suffered greater mortality rates and axillary lymph node frequency than lower grade patients. Therefore, the breast cancer grade (IDC grade) is a major indicator of breast cancer outcomes.

The breast cancer grade indicates the tumour’s aggressiveness^12^. Specifically, pathologists categorize breast cancer using the Nottingham Grading Scheme (NGS), which assigns a grade characterized by three morphological traits of the breast cancer tissue: (1) mitotic count (the number of proliferating tumour cells), (2) nuclear pleomorphism (the overall appearance of the tumour cell), and (3) degree of tubule formation (how well the tumour cells replicate normal glands)^5^. These characteristics combine to produce a total score that indicates the presence of low-grade (grade 1), intermediate-grade (grade 2), or high-grade (grade 3) breast cancer^12^. Although manual breast cancer grading remains the gold standard for cancer diagnosis, pathologists' competence can have a considerable impact on results^13^. Inexperienced pathologists may make incorrect diagnoses^14^. Manual breast cancer grading is laborious, time-consuming, and subjective, owing to pathologists’ wide intra- and inter-observational variability^13^. Elmore et al.^15^ discovered an overall agreement of around 75.3 percent between each pathologist's investigation and the expert consensus–derived reference diagnosis. Additionally, manual grading in low magnification images is susceptible to statistical, distributional, and human errors^16^.

Automated breast cancer grading approaches have risen in popularity as computer vision technology has advanced. Previous research^17–20^ attempted to overcome the manual breast cancer grading system by combining NGS criteria with classic machine learning approaches. Nevertheless, traditional approaches are highly feature-dependent, time-consuming, and expensive to compute. On the other hand, deep learning methods improve grading efficiency while reducing human workloads^21^. Wan et al.^22^ pioneered deep learning by employing a Convolutional Neural Network (CNN) to classify breast cancer grades. Several other studies^23–25^ used a range of deep learning techniques to handle this categorization problem. These techniques, on the other hand, are robust and necessitate a large amount of computer power. Transfer learning, on the other hand, is becoming increasingly common; for example, many studies^26,27^ used transfer learning to grade breast cancer. There is a knowledge gap among these research, to our knowledge: there have been no performance comparisons of recent pre-trained state-of-the-art CNN architectures ((EfficientNetB0^28^, EfficientNetV2B0^29^, EfficientNetV2B0-21k^29^, ResNetV1-50^30^, ResNetV2-50^31^, MobileNetV1^32^, and MobileNetV2^33^). As a result, many people are unaware of how CNN structures are used in automatic IDC grading. As a result, we plan to fill a knowledge gap by providing our findings on the automated IDC grading application employing several CNN architectures ranging from simple and light-weight CNNs to complicated and heavy-weight CNNs.

The purpose of this work is to examine contemporary CNN architectures in IDC grading through the use of histopathology images. The following are the study's aims, in no particular order:

1. To review the state-of-the-art CNN architectures adopted in IDC grading.
2. To conduct a comparative investigation of the performance of seven selected cutting edge CNN architectures on the Four Breast Cancer Grades (FBCG) Dataset^26^.

Our work studied seven types of CNN architectures (EfficientNetB0, EfficientNetV2B0, EfficientNetV2B0-21 k, ResNetV1-, ResNetV2-50, MobileNetV1, and MobileNetV2) in the application of automated IDC grading. We employed the transfer learning technique that leverages pre-trained CNNs from the TensorFlow Hub (TF Hub) for visual feature extraction. The saved CNNs were trained on the ImageNet dataset. We applied our proposed technique to the Four-Breast-Cancer-Grades (FBCG) dataset. Conversely, our work was accomplished without improving the pre-trained CNN architectures and implementing the effect of stain normalisation. We summarise our contributions as below:

1. We conducted a performance analysis of seven CNN architectures on IDC grading applications based on the Four Breast Cancer Grades (FBCG) Dataset.
2. We successfully designed and conducted experiments to uncover that the EfficientNetV2B0-21 k outperformed other CNN models (balanced accuracy = 0.9666 ± 0.0185, macro precision = 0.9646 ± 0.0174, recall = 0.9666 ± 0.0185 and F1 score = 0.9642 ± 0.0184 on fivefold stratified cross-validation (CV), balanced accuracy = 0.9524, macro recall = 0.9524) with only low FLOPs (0.72B), parameters (7.1 M), inference time (0.0758 ± 0.0001) and training time (0.5592 ± 0.0162).
3. We discovered that all CNN architectures exhibited comparatively good performance in IDC grading applications with an average balanced accuracy of 0.9361 ± 0.0189 (fivefold stratified CV) and 0.9308 ± 0.0211(test result).

The following is the structure of this work: Related works” section highlights the development of breast cancer grading systems. Methodology section outlines the technique used to compare the performance of seven CNN architectures. Results and discussion” section summarises our conclusions and results from the comparison study. Finally, in “Conclusion” section, we summarise our findings and discuss future developments.

## Related works

This section reviews the history of automated breast cancer grading using histopathology images. These studies are divided into two categories: classic feature-based and deep learning-based (manual feature extraction, end-to-end feature extraction, and transfer learning).

Initially, breast cancer grading was based on the NGS criteria for (1) mitotic count, (2) degree of tubule formation, and (3) nuclear pleomorphism. For example, Dalle et al.^17^ proposed a multi-resolution technique that incorporated all three NGS criteria in order to address previous automated breast cancer grading systems that only addressed portions of the NGS criteria. The proposed approach was executed in a manner comparable to manual grading. Doyle et al.^19^ suggested an automated quantitative image analysis method based on spectral clustering and image attributes from the textural and architectural domains. Prior to performing spectral clustering, the authors computed textural and architectural characteristics from the images in order to minimise the dimensionality of the feature set. The suggested technique classified low and high breast cancer grades with a 93.3% accuracy when all architectural factors were included.

Naik et al.^19^ outlined an automated gland and nuclei segmentation method for prostate and breast histopathology that integrated three types of image information: (1) low-level information based on pixel values, (2) high-level information based on the correlations between pixels for object detection, and (3) domain-specific information based on the correlations between histological structures. The proposed method achieved 80.52% and 93.33% accuracy for low and high breast cancer grades, respectively, using automated and manually extracted feature sets. Basavanhally et al.^20^ proposed a multi-field-of-view (multi-FOV) framework for grading ER + breast cancers using entire histopathology slides. The authors used a multi-FOV classifier capable of automatically integrating image features from multiple FOVs of varying sizes to predict the breast cancer grade of the images. For classifying low versus high grades, low versus intermediate grades, and intermediate versus high grades, the approach achieved area under curve (AUC) values of 0.93, 0.72, and 0.74. Dimitropoulos et al.^34^ proposed a method for automatically grading breast cancer by encoding histological images as Grassmann manifold-based Vector of Locally Aggregated Descriptors (VLAD) representations. Additionally, the authors created a new medium-sized breast cancer grading dataset. With the overlapping patch size 8 × 8 strategy, the proposed method achieved an average classification accuracy of 95.8%.

Despite their simplicity, these methods are probably obsolete in light of recent advancements in computer vision technology. Additionally, these methods are primarily feature-based, focusing exclusively on segmenting and classifying histological primitives. Additionally, these methods require a greater amount of computational power due to the complexity of the pre-processing steps (segmentation, nuclei separation, and detection) and the absence of heuristics for feature extraction ^23^.

### Deep learning based methods

Deep learning is a part of machine learning techniques inspired by the human brain to recognize patterns. Deep learning approaches train on hierarchical representations to achieve high performance. Prior domain knowledge is inessential since these methods can extract and categorize distinct features. Contrarily, conventional machine learning approaches require hand-crafted feature extraction. Hence, deep learning techniques, particularly CNNs, have become the de facto standard for medical image classification^35^. CNN is a type of deep neural network (DNN) that relies on the correlation of neighbouring pixels. Initially, CNN utilizes randomly specified patches for input and then changes the patches during model training. Subsequently, the CNN utilizes these modified patches to predict the validation and testing sets after model training. CNNs have wildly succeeded in image recognition problems as automatic feature extractors since CNNs excel in matching the data point distribution in the image. A CNN architecture comprises two types of transformations: (1) convolution layer (pixels are convolved with a filter, delivering the dot product between the image patch and filter); and (2) subsampling layer (max, min, or average pooling, functions to lower the data dimensionality). The filter dimension (height x width x depth) and the pooling filter size can be configured based on the network or user requirement. After utilizing a combination of convolution and pooling layers, the output is passed through to a fully connected layer for final classification^36^.

#### Manual feature extraction

Wan et al.^22^ proposed a method for grading breast cancer in histopathological images by combining multi-level image features at three levels: (1) pixel-level, (2) object-level, and (3) semantic-level features. The method achieved a 92% accuracy difference between low and high grades, a 77% difference between low and intermediate grades, a 76% difference between intermediate and high grades, and a 69% difference between all breast cancer grades. The multi-level features allow for accurate morphological classification of cancer while also extracting structural information and interpretable high-level concepts from histopathological images. Additionally, the use of cascaded ensembles lowers computational costs. However, the dataset used is relatively small (106 images). The implemented CNN architecture is inefficient, resulting in a lengthy training period (20 h). As a result, we intend to investigate deep learning methods that incorporate automatic feature extraction.

#### Automatic feature extraction

Li et al.^24^ proposed a multi-task deep learning method for breast cancer grading that embeds contrastive constraint as well as classification constraint (SoftMax) in the feature representation learning process. In the representation learning process, the authors combined classification and verification tasks of image pairs. The variances in feature outputs were calculated for different subclasses and within the same subclass. For the breast cancer grading task, the proposed method achieved 93.01% accuracy. Yan et al.^37^ proposed a nuclei-aware network (NANet) that grades breast cancer in histopathological images with medical intent (attention to nuclei-related features) while learning image feature representations in their entirety. The NANet is divided into two branches: (1) the main branch extracts the feature representation of the entire image, and (2) the guide branch extracts only the feature representation of the segmented nuclei image. In terms of overall breast cancer grading, the proposed model achieved 92.2% accuracy. Senousy et al.^23^, in contrast to Yan et al.^37^, proposed an Entropy-Based Elastic Ensemble of deep convolutional network (CNN) models (3E-Net) for breast cancer grading. The proposed method employs multiple CNNs as well as an ensemble-based uncertainty-measure component that selects the most certain image-wise models for the final breast cancer grading. The proposed models' two variations achieved grading accuracy of 96.15% and 99.50%, respectively. Despite their success, CNN deep learning approaches require much computational power and are more complicated than transfer learning techniques. As a result, we intend to research transfer learning techniques in IDC grading applications.

#### Transfer learning methods

CNNs with transfer learning techniques have become more prevalent in classification tasks. Numerous contemporary approaches make use of fine-tuning to enhance performance^38^. Transfer learning enhances performance by transferring knowledge from a target domain to a source domain. Hence, the dataset required for training in the target domain can be reduced^39^. Zavareh, Safayari, and Bolhasani^27^ proposed a method for classifying the Databiox^40^ using transfer learning (BCNet). The BCNet is composed of three main components: (1) a VGG16 pre-trained model that acts as a feature extractor, (2) a global average pooling layer, and (3) three dense layers that are fully connected. The BCNet achieved a validation accuracy of 88% and a test accuracy of 72% for breast cancer grading. Similarly, Abdelli et al.^26^ proposed using transfer learning to grade breast cancer using two distinct types of CNN architectures. In three breast cancer grade datasets, the MobileNetV1 achieved 93.48% accuracy, while the ResNetV1-50 achieved 92.39% accuracy. Additionally, the authors developed a novel dataset strategy (Four-Breast-Cancer-Grades Dataset) by combining two distinct breast cancer datasets to create a new class (grade 0) for breast cancer grading. Both models performed better on the new dataset than on the original dataset; the ResNetV1-50 achieved a higher accuracy of 97.03% than the MobileNetV1.

We discovered that transfer learning studies^27,28^ lack comparisons of recent pre-trained state-of-the-art CNN architectures' accuracy, complexity, size, inference time, and training time. As a result, users lack an understanding of how the CNN architecture is used in automated IDC grading. As a result, we intend to compare the performance of seven distinct types of CNN architectures for IDC grading applications.

## Summary

Early breast cancer research^17–19^ is feature-dependent, requires increased computational power, and lacks feature extraction heuristics. Deep learning methods (CNN) have evolved exponentially in recent years to excel at histopathological image analysis of breast cancer. Additionally, several studies^23,24,37^ demonstrated that deep learning methods could achieve near-perfect performance in grading breast cancer, on par with state-of-the-art approaches. Transfer learning techniques have become more prevalent in deep learning approaches, owing mainly to the presence of small datasets in breast cancer datasets. Abdelli et al.^26^ and Zavareh, Safayari, and Bolhasani^27^ used transfer learning to grade histopathological images of breast cancer. The details of these works are summarised in Table 1. However, we discovered that these publications omit performance evaluations of contemporary CNN architectures. As a result, we intend to conduct a comparative analysis of the performance of seven distinct CNN architectures used in IDC grading applications. The methods and datasets used in previous studies on breast cancer grading are summarised in Table 1. The following Table 2 summarises the available databases of breast cancer histological images.

**Table 1:** This table summarises the methods and datasets adopted by previous studies on breast cancer grading.

References	Methods	Datasets	Result
^17^	The multi-resolution method that combined the three NGS evaluation criteria and Gaussian model functions	Own Custom dataset	Quantitative results were not available Grading result was similar to the pathologists’ scores but slightly lower in general
^18^	Spectral clustering with image textural and architecture features	Own Custom dataset	93.3% accuracy with all architecture features
^19^	Segmentation method that utilised the combination of low-level, high-level, and domain specific information	Own Custom dataset	80.52% accuracy in automated feature extraction set low vs high grades 93.33% accuracy in manual feature extraction set low vs high grades
^20^	Multi field-of-view (multi-FOV) classifier	Own Custom dataset	AUC values: 0.93 (low vs high grades), 0.72 (low vs intermediate grades), 0.74 (intermediate vs high grades)
^34^	Grassmann manifold	BreaKHis and Breast Cancer Grading Dataset	95.8% accuracy (overlapping )patch size 8 × 8 strategy
^22^	Deep learning with manual feature extraction -Cascaded ensemble method with multi-level image features combination (pixel, object, semantic)	Own Custom dataset	92% (low vs high) 77% (low vs intermediate) 76% (intermediate vs high) 69% (overall)
^24^	Deep learning with automatic feature extraction -Multi-task deep learning method	BreaKHis and Breast Cancer Grading Dataset	93.33% accuracy in manual feature extraction set low vs high grades
^37^	Deep learning with automatic feature extraction Nuclei aware network (NaNet) that applies more attention into nuclei related features while learning the whole pathological image feature representation	Breast Cancer Grading Dataset with own custom dataset	92% for overall IDC grading
^23^	Deep learning with automatic feature extraction Entropy-Based Elastic Ensemble of deep convolutional network (CNN) models (3E-Net) for breast cancer grading	BreaKHis and Breast Cancer Grading Dataset	3E-Net (Version A): 96.15% accuracy 3E-Net (Version b): 99.50%,
^26^	Transfer learning (feature extraction) using ResNetV1-50 and MobileNetV1	BreaKHis and Breast Cancer Grading Dataset	Four Breast Cancer Grade dataset: 97.03% accuracy (ResNet50), 94.42% accuracy (MobileNet) Three Breast Cancer Grade dataset: 92.39% accuracy (ResNet50), 93.48% accuracy (MobileNet)
^27^	Transfer learning (feature extraction) using VGG16	Databiox	88% validation accuracy 72% test accuracy

**Table 2:** This table summarises available databases of breast cancer histological images.

Dataset	Format	Number of Images	Classes	Resolutions	Magnification
IDC dataset^41^	RGB	162 277,524 non-overlapping patches	IDC positive and IDC negative	Patch size: 50 × 50	40×
Bioimaging 2015 dataset^42^	RGB	249	Normal, benign, in situ and carcinoma	2048 × 1536	200×
ICIAR2018 ^38^	RGB.tiff	400	Normal, benign, in situ and carcinoma	2048 × 1536	200×
BreaKHis^43^	RGB	7909	Benign (2480 images) and Malignant (5429 images)	700 × 460	40×, 100 × ,200 × , and 400 ×
Breast Cancer Grading Dataset^44^	RGB	300	Grade 1 (107 images), Grade 2 (102 images) and Grade 3 (91 images)	1280 × 960	40×
Databiox^45^	RGB, JPEG	922	Grade 1 (259 images), Grade 2 (366 images) and Grade 3 (297 images)	2100 × 1574, 1276 × 956	4×, 10×, 20 × and 40×

## Methodology

**Figure 1:**
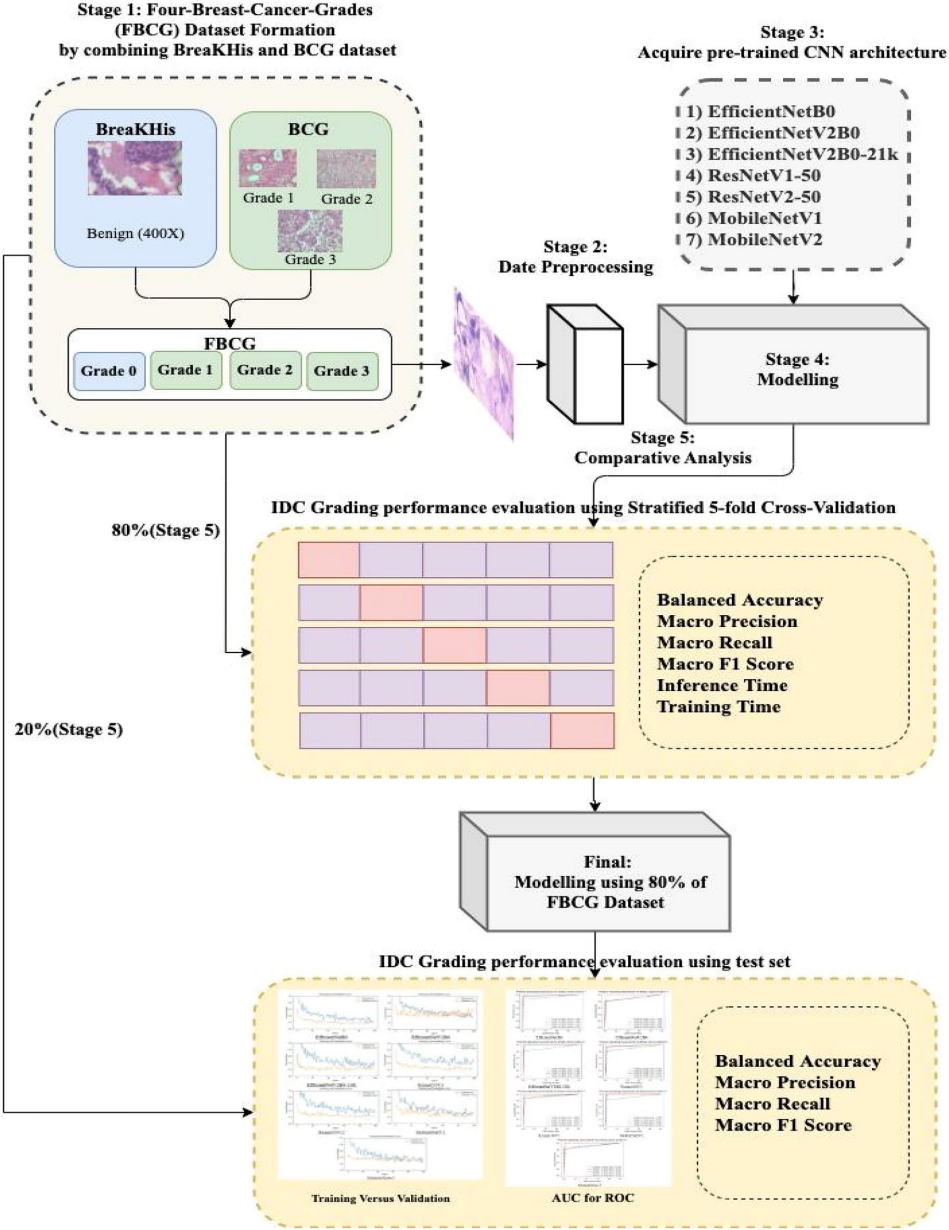
This figure shows the overall flow of our methodology. First, a four-grade dataset (termed the "Four Breast Cancer Grades (FBCG) dataset") is established using BreaKHis and BCG datasets. The selected seven pre-trained CNN architectures are used to model 80% of the FBCG using a fivefold stratified CV approach on the pre-processed data. After confirming the stability of all the models via CV, a final model is trained using all the training data. The final model is evaluated using a test dataset (the 20% of FBCG). The receiver operating characteristics curves and training versus validation curves are used to compare and analyse the performance of all the models that are chosen.

In this section, we described the methodology for the comparative analysis of the performance of 7 CNN architectures in IDC grading applications using pre-trained CNNs from the TF Hub for image feature extraction (transfer learning). We adopted the Four-Breast-Cancer-Grades (FBCG) Dataset. We fed the datasets into our proposed method that utilised the seven different pre-trained CNN architectures for feature extraction. Our experiments were conducted on the Google Collaboratory platform, which meets the following specifications: (1) 2.30 GHz Intel(R) Xeon(R) CPU, (2) 12 GB RAM, (3) up to 358 GB disc space, and (4) 12 GB/16 GB Nvidia K80/T4 GPU. For our work, we primarily used the TensorFlow library. Our approach is divided into four stages: (1) image data pre-processing, (2) custom CNN construction (using pre-trained CNNs from TF Hub as feature extractor), (3) model compilation and training, and (4) model evaluation. The stages of our methodology are summarised in Fig. 1. We confirm that all procedures were carried out in accordance with relevant guidelines and regulations.

### Dataset

**Figure 2:**
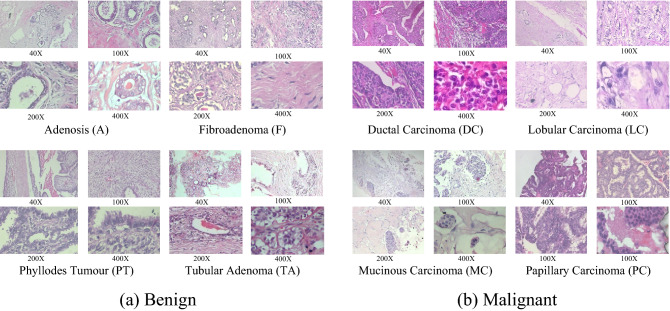
Samples slides of different breast tumour types (stained with H&E) under 40X,100X,200X, and 400X magnification factors from BreaKHis for two tumour classes: (**a**) benign, (**b**) malignant. Our research considers all histological images from the Benign class as “Grade 0”.

The FBCG dataset comprises two datasets: (1) BreaKHis^43^ and (2) the Breast Cancer Grading (BCG) dataset^44^. BreaKHis contains 7909 histopathological images of breast cancer obtained from 82 patients at four different magnification factors (40X, 100X, 200X, and 400X), corresponding to four different objective lenses (4X, 10X, 20X, and 40X). The dataset is primarily divided into two categories: benign (2480 images) and malignant (5429 images); benign and malignant breast tumours can be further classified into four distinct types: Adenosis (A), Fibroadenoma (F), Phyllodes Tumour (PT), and Tubular Adenoma (TA) for the benign class; and Ductal Carcinoma (DC), Lobular Carcinoma (LC), Mucinous Carcinoma (MC), and Pa (see Fig. 2). The term "benign" has historically been used to refer to a lesion that lacks malignant characteristics such as metastasis (spreading from an initial site to a secondary site), significant cellular atypia (appears abnormal in shape, colour, or size), mitosis (parent cells divide and grow), and disruption of basement membranes (which are the thin, dense sheets of the specialised extracellular matrix that surround tissues). In general, benign lesions are non-aggressive, growing slowly, with distinct borders, and remaining localised. Malignant lesions are frequently locally invasive and have a proclivity to invade distant sites, resulting in death. The images were created using Hematoxylin and Eosin (H&E) stained breast tissue biopsy slides and then processed into a digital RGB format with a resolution of 700 × 460 pixels. The BreaKHis is summarised in Table 2. The distribution of images by class and magnification factor is shown in Table 3.

**Table 3:** This table illustrates the image distribution of BreaKHis by class and magnification factor.

Magnification	Benign	Malignant	Total
40x	625	1370	1995
100x	644	1437	2081
200x	623	1390	2013
400x	588	1232	1820
Total	2480	5429	7909

**Figure 3:**
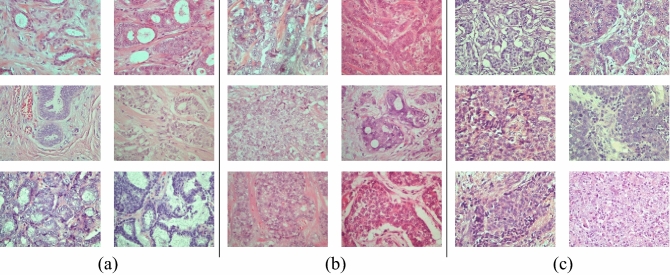
Random samples from each grade in the BCG dataset: (**a**) Grade 1, (**b**) Grade 2, (**c**) Grade 3.

Zioga et al.^44^ published the BCG dataset containing different grades of breast cancer histological images. Each breast carcinoma histological sample was collected in the Department of Pathology at Thessaloniki's "Agios Pavlos" General Hospital, Greece, using a Nikon digital camera equipped with a 40X objective lens (equivalent to a magnification of 400X in the BreaKHis dataset). This dataset contains 300 images with a resolution of 1280 × 960 and staining with H&E. The dataset contains three IDC grades (107 images), grade 2 (102 images), and grade 3 (91 images) that correspond to 21 patients based on their NGS results: grade 1 (107 images), grade 2 (102 images), and grade 3 (91 images) (see examples in Fig. 3).

The FBCG dataset^26^ is created to address the constraints associated with small breast cancer datasets. The FBCG dataset is formed by combining the magnified 400X benign images (as Grade 0) from the BreaKHis with the Grade 1, 2, and 3 images from the BCG dataset. For the experiments, the dataset was divided into a 20% test set and an 80% training set with no overlap. The test set images were chosen through stratification (the first portion of images in the dataset was selected to form the test set). The distribution of images in the FBCG dataset is summarised in Table 4.

**Table 4:** This table shows the image distribution of the FBCG dataset.

		Grade 0	Grade 1	Grade 3	Grade 3	Total
FBCG dataset	Train set	470	86	82	73	711
	Test set	118	21	20	18	177
	Total	588	107	102	91	888

### Data pre-processing

Pre-processing the data is critical for converting it to a format compatible with the pre-trained CNN architectures. To perform the fivefold stratified CV, we divided the training set into five folds. Stratified fivefold CV ensures that each training set fold obtains the same proportion of observations with a given label while ensuring that each CNN model is properly trained. The "ImageDataGenerator" class (from Keras pre-processing.image) was used to normalise the images by scaling them by 1/255. (original images are composed of RGB coefficients ranging from 0 to 255, which are incompatible with CNN models). Then, using the "flow_from_dataframe" method, we applied image normalisation to the training set using the configurations listed in Table 6. The FBCG dataset's image sizes (700 × 460 and 1280 × 960) are large in comparison to the CNN models' input sizes (see Table 5). We noticed that resizing images preserved global characteristics but ignored local characteristics. As a result, the model's performance would be highly dependent on the model's ability to recognise and learn global features^46^.

**Table 5:** This table summarises the seven CNN architectures adopted for the comparative analysis in terms of their main contributions, datasets involved, FLOPs, parameters and input shapes.

Architectures	Main contributions	Datasets	FLOPs (B)	Parameters (M)	Input shapes
EfficientNetB0^28^	Compound scaling	ImageNet-ILSVRC-2012-CLS)^47^	0.39	5.3	224 × 224
EfficientNetV2B0^29^	Progressive learning	ImageNet-ILSVRC-2012-CLS	0.72	7.1	224 × 224
EfficientNetV2B0-21k^29^	Progressive learning	ImageNet-21k^48^	0.72	7.1	224 × 224
ResNetV1-50^49^	Residual learning	ImageNet-ILSVRC-2012-CLS	4.1	25.6	224 × 224
ResNetV2-50^31^	Identity mapping	ImageNet-ILSVRC-2012-CLS	4.1	25.6	224 × 224
MobileNetV1^32^	Depth-wise separable convolutions	ImageNet-ILSVRC-2012-CLS	0.6	4.2	224 × 224
MobileNetV2^33^	Inverted residuals and linear bottlenecks	ImageNet-ILSVRC-2012-CLS	0.3	3.4	224 × 224

### Data augmentation

Data augmentation is a standard procedure to address the risk of model overfitting during model training by increasing the number of input images of the dataset^50^. This procedure also assures a fairer comparison between our study results and other published results in the literature. Although The FBCG dataset contains 888 images, the dataset is still considered small relatively; as a result, model overfitting may occur during model training. Thus, we implemented data augmentation by infusing the training samples with artificial diversity via random but realistic transformations. We used the TensorFlow Keras pre-processing layers to augment the data. The data augmentation layers supplement the training data but are disabled during validation and testing operations. We used three techniques for augmentation: (1) random horizontal and vertical flips, (2) random rotation, and (3) random zoom (see Table 6). We used random flipping and rotation because pathologists' ability to examine histopathological images is not affected by rotation angles. As a result, we assumed that different rotation angles would not affect the CNN's ability to learn. Additionally, we used random zoom augmentation to simulate the magnification factor found in histopathological images of breast cancer in order to enhance the CNN's generalisation ability.

**Table 6:** This table summarises the pre-processing, data augmentation, and model compilation details for the standardised framework.

	Parameters	Values
Pre-processing (flow_from_dataframe)	target_size	N × N (see Table 5)
	batch_size	16
	shuffle	True
	seed	123
	class_mode	categorical
Data Augmentation	RandomFlip	horizontal_and_vertical
	RandomRotation	0.2
	RandomZoom	0.2
Model Compilation	Optimiser	Adam Optimiser
	Learning rate	0.001
	Loss function	Weighted Categorical Cross Entropy
	Metrics	Accuracy
	Epochs	100

### Data balancing

The FBCG data set is imbalanced (see Table 4). An imbalanced dataset will cause the CNN model to be more biased toward predicting the majority class. We used the class weighting technique from the Scikit-Learn Python library to resolve this concern. This technique grants the minority class a higher weight in the model cost function in order to impose a greater penalty on the minority class. As a result, the model can converge on the objective of minimising errors for the minority class^51^. We used the following equation to determine the weight of each class:

1 $$W = \frac{N}{{N_{c} \times N_{sc} }}$$

where $W=$ class weight. $N=$ total number of samples. ${N}_{c}=$ number of classes. ${N}_{sc}=$ number of samples in each class.

### Transfer learning

CNN approaches only perform well when the models are trained on large and well-annotated datasets. Nevertheless, the FBCG dataset is considered small (888 images). Therefore, we opted for the CNN with transfer learning technique to address the issue of small datasets (model overfitting). Additionally, transfer learning can reduce model training time and improve model performance^39^. Transfer learning consists of four components: (1) source domain (${\mathrm{D}}_{\mathrm{s}}$), (2) target domain (${\mathrm{D}}_{\mathrm{t}}$), (3) source learning task (${\mathrm{T}}_{\mathrm{s}}$), and (4) target learning task (${\mathrm{T}}_{\mathrm{t}}$); transfer learning attempts to improve the target predictive function ${\mathrm{D}}_{\mathrm{t}}(.)$ in ${\mathrm{D}}_{\mathrm{t}}$ with the knowledge in ${\mathrm{D}}_{\mathrm{s}}$ and ${\mathrm{T}}_{\mathrm{s}}$, where ${\mathrm{D}}_{\mathrm{s}}\ne {\mathrm{D}}_{\mathrm{t}}$ or ${\mathrm{T}}_{\mathrm{s}}\ne {\mathrm{T}}_{\mathrm{t}}$^25^. Generally, the first few layers of a CNN recognise more generic features (edges and generic shapes), whereas the final few layers recognise problem-specific features. Thus, transfer learning utilises of the general features learned in the first few layers of the source dataset and then relearns the specific features of the target dataset in the final few layers. Since the first few layers’ features still remain relevant to the problem, transfer learning makes the model training process fast and reduces the amount of data required for model training^39^. Therefore, transfer learning enables small datasets to be trained on CNN models with minimal risk of model overfitting.

#### Transfer learning techniques

Transfer learning entails two distinct methods for customising a pre-trained model:

1. Feature Extraction; this technique leverages a previous network's representations to extract critical features from a new dataset. This is accomplished by superimposing new classifier layers (that have been trained from scratch) on top of the pre-trained model (no training required). As a result, previously learned feature representations can be repurposed for the new dataset.
2. Fine-tuning; this technique unfreezes several top layers of a frozen base model (pre-trained model) and then trains the newly added classifier layers along with the unfrozen base model layers. This process "fine-tunes" the base model's specific feature representations (high-order features) to make the representations more applicable for the particular task^52^.

While fine-tuning the model may improve performance, this technique may induce overfitting. To avoid overfitting, we utilised seven pre-trained CNN architectures (EfficientNets^28,29^, ResNets^30,31^ and MobileNets^32,33^) as feature extractors in this work. Early CNN architectures (LeNet^53^, AlexNet^54^, and GoogleNet^55^) were disregarded as they were considered outdated and no longer state-of-the-art. Hence, comparing more recently developed models is more meaningful and inclusive. We utilised each pre-trained CNN architecture in the form of an image feature vector (a dense 1D tensor describing the whole image), reposited in the TF Hub. To apply the feature vector to our work, we employed the "hub.KerasLayer" to integrate the feature vector into our framework. This layer produces a batch of feature vectors whose size is proportional to the input size. The comparison of the seven CNN architectures is summarised in Table 5.

### Experimental details

We constructed the IDC grading model using the Keras Functional API by combining data augmentation (described in the Data Augmentation Section), pre-trained CNN architectures (feature vector), and several new classifier layers. Thus, the final IDC grading model is composed of seven layers: (1) an input layer, (2) a data augmentation layer, (3) the feature vector, (4) a dropout layer with a rate of 0.5, (5) a dense layer of 256 neurons with ReLU activation, (6) a dropout layer with a rate of 0.4, and (7) a dense layer of four neurons with the SoftMax activation function (*N* = number of classes).

#### Standardizing model pipelines and hyperparameters

**Figure 4:**
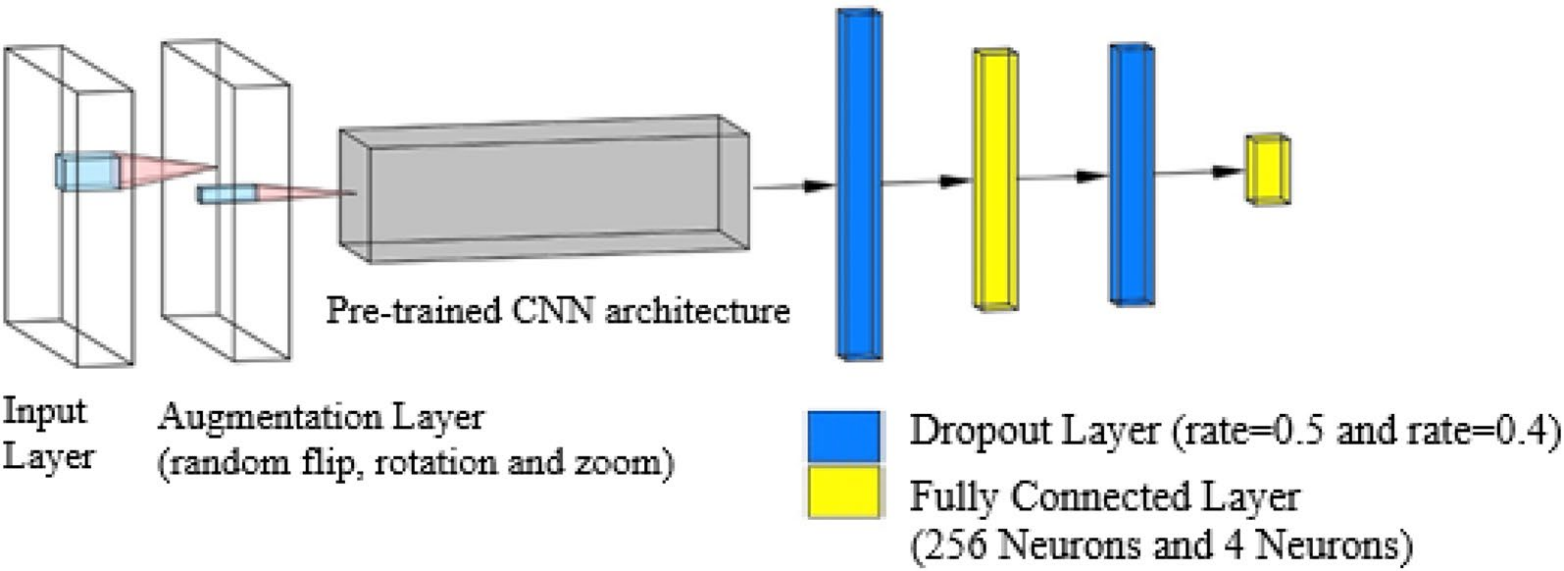
This figure illustrates the standardised pipelines for comparison purposes. The grey box represents one of the seven CNN architectures. Table 6 contains the details of the standardised framework and hyperparameters.

We standardised the model pipelines and hyperparameters to ensure fair comparisons. Munien and Viriri^46^ were the inspiration for the standardised framework. Initially, the input layer assigned a specific shape to the input data (image resolution). Then, during model training, the data augmentation layer augmented (randomly flips, rotates, and zooms) the input data. Subsequently, the input data was fed into a pre-trained CNN model (feature vector) to extract features. The output data was then passed through a first dropout layer with a rate of 0.5, a fully connected layer with 256 neurons, a second dropout layer with a rate of 0.4, and an output fully connected layer (4 neurons). If the input units were not set to 0, they were scaled up by 1/(1 − rate) to maintain the same sum of all inputs^56^. Finally, the dense layer's SoftMax function converted the model output to a vector of probabilities for each class's input data. The architecture of our proposed framework is depicted in Fig. 4.

#### Model compiling

We adopted the Adam Optimiser with a learning rate of 0.001. Determining an appropriate learning rate is critical for model training since it affects the time required for the model to converge to local minima. A rapid rate of learning may induce the model to deviate from its local minima. On the other hand, a slow learning rate may impede model training, resulting in increased computational costs^57^. Thus, we chose the 0.001 learning rate as the optimal value after undertaking several empirical tests. Correspondingly, we implemented the weighted categorical cross-entropy loss function for the classification task that required the use of the weight class technique and the metrics parameter "accuracy." Finally, each fold was trained for 100 epochs. The details of the model's construction are summarised in Table 7. The weighted categorical cross-entropy loss function is described as:

2 $${\text{WCE}} = - w_{j} *log\left( {\frac{{e^{{s_{p} }} }}{{\mathop \sum \nolimits_{j}^{c} e^{{s_{j} }} }}} \right)$$

**Table 7:** This table summarises the results acquired from the fivefold stratified CV.

	Balanced accuracy	Macro precision	Macro recall	Macro F1 score	Kappa score	Inference time (s)	Training time (h)
EfficientNetB0	0.9303 ± 0.0322	0.9161 ± 0.0408	0.9303 ± 0.0322	0.9211 ± 0.0378	0.9180 ± 0.0362	0.0810 ± 0.0006	**0.5565 ± 0.0088**
EfficientNetV2B0	0.9076 ± 0.0398	0.8988 ± 0.0429	0.9076 ± 0.0398	0.9000 ± 0.0416	0.9040 ± 0.0455	0.0753 ± 0.0004	0.5630 ± 0.0287
EfficientNetV2B0-21k	**0.9666 ± 0.0185**	**0.9646 ± 0.0174**	**0.9666 ± 0.0185**	**0.9642 ± 0.0184**	**0.9678 ± 0.0154**	0.0758 ± 0.0001	0.5592 ± 0.0162
ResNetV1-50	0.9253 ± 0.0310	0.9244 ± 0.0358	0.9253 ± 0.0310	0.9206 ± 0.0334	0.9255 ± 0.0313	0.2184 ± 0.0014	0.5795 ± 0.0556
ResNetV2-50	0.9346 ± 0.0156	0.9199 ± 0.0276	0.9346 ± 0.0156	0.9259 ± 0.0202	0.9233 ± 0.0247	0.2277 ± 0.0010	0.5968 ± 0.0478
MobileNetV1	0.9518 ± 0.0232	0.9526 ± 0.0180	0.9518 ± 0.0232	0.9506 ± 0.0214	0.9543 ± 0.0181	**0.0424 ± 0.0004**	0.5628 ± 0.0340
MobileNetV2	0.9362 ± 0.0322	0.9339 ± 0.0251	0.9362 ± 0.0322	0.9314 ± 0.0305	0.9357 ± 0.0278	0.0456 ± 0.0011	0.5659 ± 0.0818

Each performance metric was reported in average (± standard deviation) form. The bold values represent the best score in each category. The best overall performing model was found to be EfficientNetV2-B0-21k, and the fastest CNN model was MobileNetV1.

where $S_{p} =$ positive output score. $S_{j} =$ other classes output scores. ${\text{WCE}} =$ weighted categorical cross entropy. $w_{j} =$ classes weights.

### Performance evaluation metrics

We used the macro-average technique to evaluate the precision, recall, and F1 score of the seven CNN architectures due to data imbalance. The macro-average method calculates each class metric independently and then averages the results, ensuring that all classes are treated equally. For the accuracy score, we used the balanced accuracy score from Scikit-Learn to calculate the average recall per class. The inference time indicates the average amount of time required for the CNN model to predict a single image. The training time is the period required for the CNN model to complete 100 epochs of training. Finally, we quantified the model's ability to distinguish between classes using the Area Under the Receiver Operating Characteristic (ROC) Curve (AUC) ^58^. The following mathematical expressions define the evaluation metrics:

3 $${\text{Balanced Accuracy}} = \frac{1}{\left| G \right|}\mathop \sum \limits_{i = 1}^{\left| G \right|} \frac{{TP_{i} }}{{TP_{i} + FN_{i} }}$$

4 $${\text{Precision }}_{{{\text{macro}}}} = \frac{1}{\left| G \right|}\mathop \sum \limits_{i = 1}^{\left| G \right|} \frac{{TP_{i} }}{{TP_{i} + FP_{i} }}$$

5 $${\text{Recall }}_{{{\text{macro}}}} = \frac{1}{\left| G \right|}\mathop \sum \limits_{i = 1}^{\left| G \right|} \frac{{TP_{i} }}{{TP_{i} + FN_{i} }}$$

6 $$F1_{{{\text{macro}}}} = 2\frac{{{\text{Precision}}_{{{\text{macro}}}} \times {\text{Pecall}}_{{{\text{macro}}}} }}{{{\text{Precision}}_{{{\text{macro}}}} + {\text{Precision}}_{{{\text{macro}}}} }}$$

7 $${\text{Inference }}\;{\text{time}} \left( s \right) = \frac{1}{10}\mathop \sum \limits_{i = 1}^{n = 10} \left( {\frac{{T_{f} - T_{in} }}{{N_{s} }}} \right)_{i}$$

8 $${\text{Training }}\;{\text{Time}} \left( h \right) = \frac{1}{3600}\mathop \sum \limits_{i = 1}^{n = 100} (T_{t} )_{i}$$

where

$$G = \left\{ {1, \ldots ,4} \right\} \left( {Number\, of \,classes} \right)$$

$TP =$ true positive. $TN =$ true negative. $FP =$ false positive. $FN =$ false negative. $T_{f} =$ final prediction time for all the images in the validation/test set. $T_{i} =$ initial prediction time for all the images in the validation/test set. $N_{s} =$ number of validation/test samples. $T_{t} =$ training time.

## Summary

In summary, we used the FBCG dataset to compare the performance of seven different CNN architectures. Our approach was divided into four stages: (1) image data pre-processing, (2) custom CNN construction (using pre-trained CNNs from TF Hub as feature extractor), (3) model compilation and training, and (4) model evaluation. We divided the dataset into 80% training and 20% test sets (see Table 4). The training set was then subjected to the fivefold stratified CV. To pre-process our dataset, we used the "ImageDataGenerator" class and the "flow_from_dataframe" method (see Table 6). Additionally, we used TensorFlow Keras pre-processing layers to augment the data (see Table 6). We implemented the Scikit-Learn Python library's class weighting technique for the unbalanced data. To classify the FBCG dataset, we used seven pre-trained CNN architectures as feature extractors (see Fig. 4 for model framework; see Table 6 for model compiling). Finally, we evaluated each CNN architecture's performance using the following metrics: balanced accuracy, macro precision, macro recall, macro F1 score, inference time, and training time.

## Results and discussion

We classified the FBCG dataset into four grades using selected state-of-the-art pre-trained CNN architectures (EfficientNetB0, EfficientNetV2B0, EfficientNetV2B0-21k, ResNetV1-50, ResNetV2-50, MobileNetV1, and MobileNetV1). Table 7 summarises the performance metrics (balanced accuracy, macro precision, macro recall, macro F1-score, inference time, and training time) of each CNN architecture obtained from the fivefold stratified CV. CV was performed on all the training images to assure the stability of the model (For the test set result, see Table 8). The EfficientNetV2B0-21k yielded the highest balanced accuracy score (0.9666 ± 0.0185), macro precision (0.9646 ± 0.0174), recall (0.9666 ± 0.0185) and F1 score (0.9642 ± 0.0184) among the other CNN models. The high performance of the EfficientNetV2-B0-21k may be attributable to the pre-trained ImageNet21k dataset. The ImageNet21k dataset comprises approximately 12.4 million images, which is larger and more diverse than the previous ImageNet1k. The authors claimed that the pre-training on ImageNet21k outperformed the pre-training on ImageNet1k^48^.

**Table 8:** Breast cancer grading results on the test set using the final retrained model (using all training images).

Model	Balanced accuracy	Macro precision	Macro recall	Macro F1-score
EfficientNetB0	0.9518	0.9511	0.9518	**0.9494**
EfficientNetV2B0	0.9024	0.9046	0.9024	0.8982
EfficientNetV2B0-21k	**0.9524**	**0.9524**	**0.9524**	0.9484
Resnet50V1	0.9239	0.9169	0.9239	0.9175
Resnet50V2	0.9198	0.9012	0.9198	0.9096
MobileNetV1	0.9524	0.9545	0.9524	0.9487
MobileNetv2	0.9128	0.9028	0.9128	0.9058

The EfficientNetV2B0-21k aligns with the CV performance result (Table 7), remaining as the CNN model with the highest balanced accuracy, macro precision, and macro recall. Similarly, the MobileNetV1 achieved the highest balanced accuracy, macro precision, and macro recall, placing it as the second-best CNN model. Significant values are in bold.

While MobileNetV2 failed to outperform other CNN architectures, it has the fewest FLOPs (0.3B). (the “FLOPs” here refer to the number of floating-point operations that indicate the complexity of the model architecture; the higher the number of FLOPs, the more complex the model is). Similarly, the MobileNetV1 demonstrated a trade-off between accuracy and complexity in terms of parameter count (4.2 M) and computation time (0.0424 ± 0.0004 s) (the number of parameters represents the size of the CNN model, whereas the inference time indicates the speed of the CNN model in image prediction). Additionally, the EfficientNetB0 achieved a mediocre performance metric score with the least amount of training time (0.5565 ± 0.0088 h) (the training time is the average training period acquired from the fivefold stratified CV).

In general, the EfficientNetV2B0-21k model outperformed other CNN models in terms of balanced accuracy, macro precision, recall, and F1 score while being simpler (0.72B), smaller (7.1B) and requiring less inference time (0.0758 ± 0.0001 s) and training time (0.5592 ± 0.0162 h). In comparison to other CNN architectures, the MobileNetV1 is identified as the fastest (with an inference time of 0.0424 ± 0.0004 s).

For IDC grading purposes, CNN models with greater accuracy are preferred. In order to determine the best treatment for breast cancer patients, the IDC grading classification requires high precision. Automated IDC grading is most likely deployed in a healthcare facility equipped with high-power heavyweight workstations. Thus, resource-intensive CNN models would not be a criterion for selecting the optimal CNN architectures unless the IDC grading applications are extended to real-time settings in the future. Other applications, on the other hand, such as smartphone-based skin disease classification^59,60^, breast cancer detection in mobile devices^61^, and organ segmentation applications^62–64^ necessitate compact size and low computational cost CNNs. In these applications, a lighter (fast and compact) or equipped with Minimum Redundancy Maximum Relevance (mRMR) CNN approaches^21,65^ that can reduce computational time and cost would be preferred over a more accurate but complex CNN architecture.

All CNN models used in the automated IDC grading application demonstrated a high degree of capability for classifying IDC grades; the EfficientNetV2B0 model achieved the lowest accuracy (0.9076 ± 0.0398), while the EfficientNetV2B0-21k model achieved the highest accuracy (0.9666 ± 0.0185). The average accuracy of the seven CNN models is 0.9361, with a standard deviation of 0.0189. The low standard deviation score indicates only a slight discrepancy between the seven CNN architectures, demonstrating that all examined CNN architectures are capable of accurately classifying IDC grades. Thus, in addition to accuracy, other factors can be considered when selecting the optimal CNN architectures for a particular IDC grading application (such as model complexity, model size and inference time). For instance, in the event of limited resources, a simpler CNN model (such as MobileNetV1) is preferred.

However, not all CNN models are equally capable of predicting IDC grades with a short inference time; MobileNetV1 took the shortest inference time (0.0424 ± 0.0004 s), while ResNetV2-50 took the longest (0.2277 ± 0.0010 s). The average time required for inference is 0.1094 ± 0.0791 s. The large discrepancy indicates that several CNN models (MobileNetV1, MobileNetV2, and EfficientNetV2B0-21k) are capable of achieving high accuracy while requiring minimal inference time. In comparison, certain CNN models (ResNetV1-50, ResNetV2-50) can achieve high accuracy only at the expense of a long inference time. Although IDC grading applications prioritise accuracy over speed, embedded systems such as the Nvidia Jetson TX1, TX2, and Raspberry Pi 3 (B +) require fast and light-weight CNN models. Real-time CNN applications^66,67^ implement embedded systems with a short inference time, low power consumption, and a small computational cost. As a result, deep learning techniques can be used to implement IDC grading applications.

**Figure 5:**
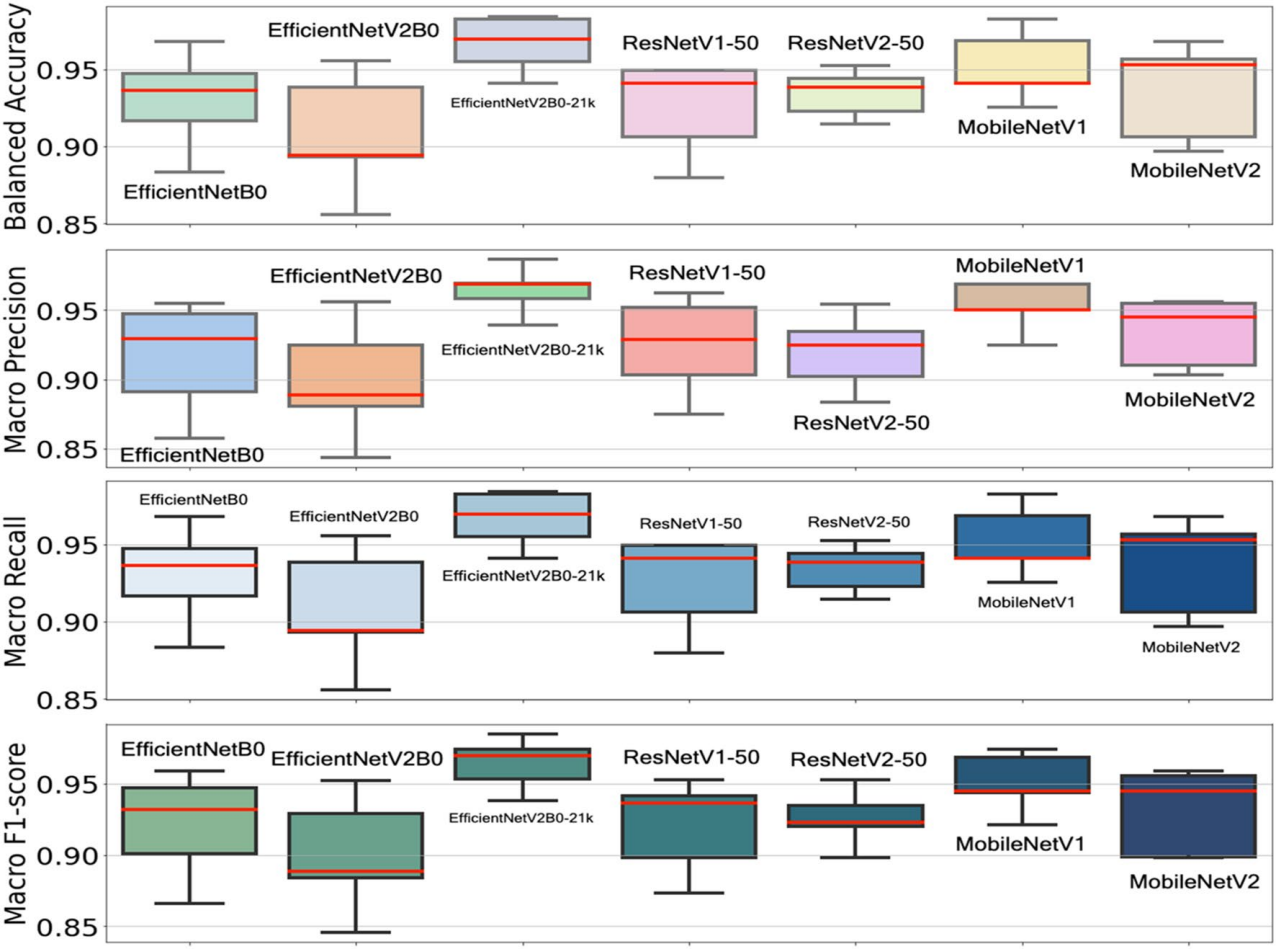
The balanced accuracy, macro precision, macro recall, and macro F1 score of seven CNN model architectures on the FBCG dataset as determined by the fivefold stratified CV are shown in this figure. The median score for each C-CNN model is indicated by the red colour lines. The EfficientNetV2B0-21k models achieved the highest maximum score in each metric, while the EfficientNetV2B0 model achieved the lowest minimum score. Except for the EfficientNetV2B0-21k and ResNetV2-50 models, the majority of CNN models scored above the 0.9 median scores, were negatively skewed (median was closer to the top quartile), and more dispersed (more dispersed data indicates more scattered data).

For balanced accuracy, precision, recall, and F1 score (median score > 0.9), all seven CNN architectures achieved high scores in Fig. 5. As a whole, these CNN models have an acceptable score range (> 0.9), except for EfficientNetB0, ResNetV1-50, and MobileNetV2. As a result of these findings, the classic CNN models (ResNetV1-50 and ResNetV2-50) are comparable to recent CNN models (EfficientNetB0 and EfficientNetV2B0s). Choosing a CNN architecture may not be the main concern in IDC grading. The user's needs should be prioritised over other factors (resource availability, hardware type, and cost).

**Figure 6:**
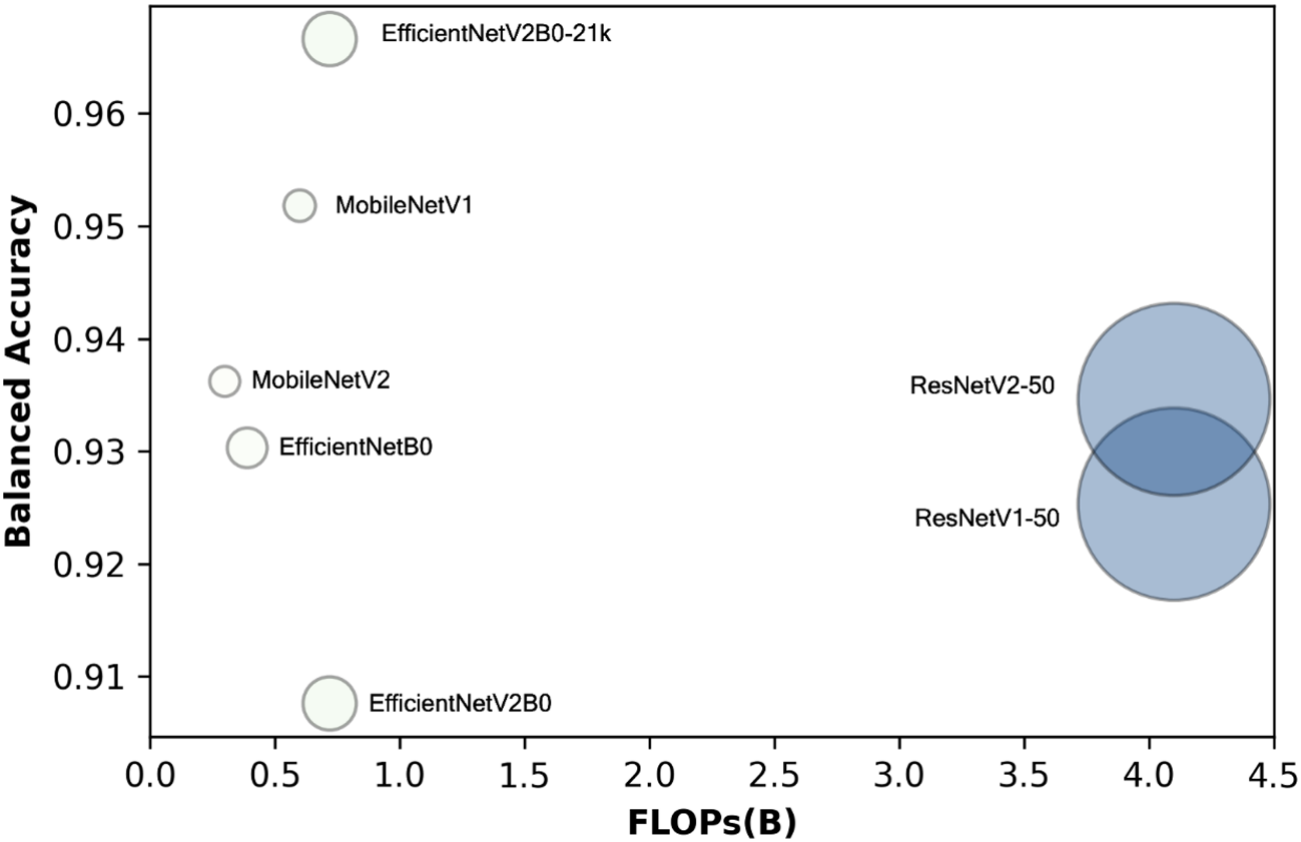
The floating-point operations per second (FLOPs) and parameters versus the balanced accuracy of the seven CNN models on FBCG dataset from the fivefold stratified CV (FLOPs in the x-axis depicts the number of operations in billions, while the radius of the circle represents the number of parameters in millions). The EfficientNetV2-B0-21k model scored the highest score (0.9666) with relatively low FLOPs (0.72B) and parameters (7.1 M). The ResNetV1-50 model achieved a low accuracy (0.9253) score with the highest FLOPs (4.1B) and parameters (25.6 M). Most of the CNN models scored average accuracy scores between 0.93 and 0.4. Generally, the average accuracy score is increasing with the FLOPs except for the EfficientNetV2B0 and ResNetV-501 and ResNetV2-50. There is no evidence that larger parameter CNN models (ResNetV1-50 and ResNetV2-50) are more accurate.

According to Fig. 6, complex and large-weight CNN models (ResNetV1-50 and ResNetV2-50) may not outperform simpler and light-weight CNN models (EfficientNetV2B0-21k, MobileNetV1, MobileNetV2). The EfficientNetV2B0-21k model achieved the highest accuracy score (0.9666) while requiring only 0.72B FLOPs and 7.1 M parameters. On the other hand, the ResNetV1-50 model achieved a low accuracy score (0.9253) despite being associated with the highest FLOPs (4.1B) and parameters (25.6 M). CNN models with a high FLOPS count do not always perform well in IDC grading applications. As a result, simpler CNN models can be used to reduce computational costs while maintaining high performance. Similarly, the scatter plot demonstrates that heavy-weight (more parameters) CNN architectures do not always outperform light-weight (fewer parameters) CNN architectures. Despite its large number of parameters (25.6 M), the ResNetV1-50 model achieved a mediocre accuracy score (0.9253). In comparison, the EfficientNetV2-B0-21k with 7.1 M parameters outperformed all other CNN models. As a result, it is more cost-effective to choose a lightweight CNN capable of producing relatively high accuracy.

**Figure 7:**
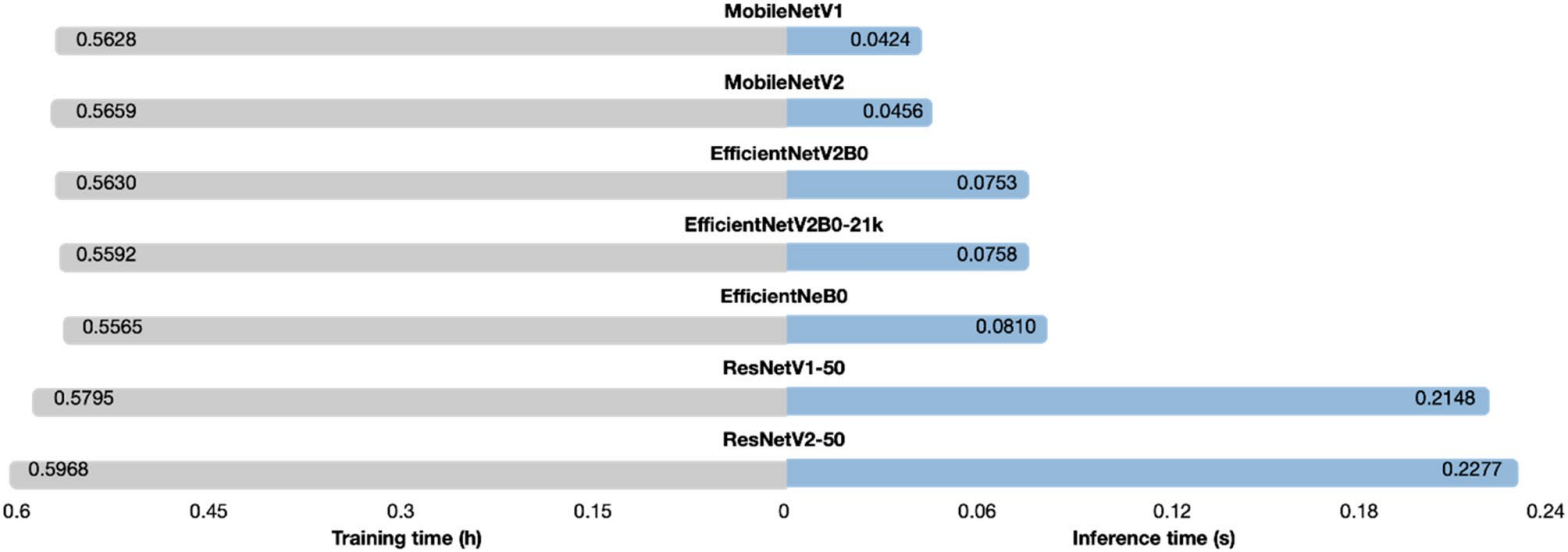
This bar chart depicts the inference time (seconds) and training time (hours) for seven CNN models trained on the FBCG dataset using the fivefold stratified CV (a low inference time indicates that the CNN model can predict the result in a short period; a low training time indicates that the CNN model can be trained in a short period). The majority of CNN models (with the exception of ResNetV1-50 and ResNetV2-50) can predict outputs in less than 0.1 s. MobileNetV1 predicts outputs the fastest (inference time = 0.0424 s), while ResNetV2-50 predicts outputs the slowest (inference time = 0.2277 s). All selected CNN models can be trained in 0.6 h.

According to Fig. 7, most CNN models (except ResNetV1-50 and ResNetV2-50) can generate predictions in less than 0.1 s. MobileNetV1 predicts outputs the fastest (inference time = 0.0424 s), while ResNetV2-50 predicts outputs the slowest (inference time = 0.2277 s). As a result, MobileNetV1 would be more suitable for real-time applications such as breast cancer detection on mobile devices^61^ and skin disease classification on smartphones^68^. However, with a short inference time (0.0758 s), the EfficientNetV2-B0-21k outperformed all CNN models (balanced accuracy = 0.9666). As a result, the EfficientNetV2-B0-21k can provide the best of both worlds (accuracy and inference time). With regards to the training time parameter, all CNN models can be trained in less than 0.6 h. ResNetV1-50 and ResNetV2-50 (heavy-weight) achieved lower accuracy at the expense of increased training time (0.5795 h and 0.5968 h). On the other hand, the EfficientNetV2B0-21k model outperformed all other CNN models (0.9666) despite requiring little training time (0.5592 h). As a result, the EfficientNetV2-B0-21k model is well-suited for applications that require high performance but require little training.

**Figure 8:**
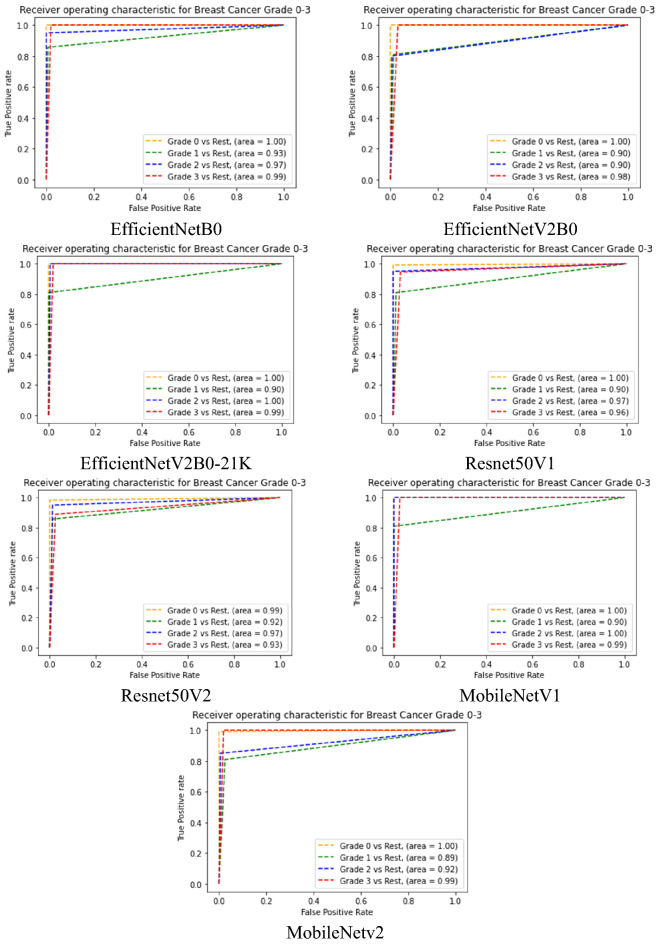
ROC curves for each of the seven chosen comparative CNN models (training set from 80% of FBCG dataset used previously for the fivefold stratified CV) on test set (20% of FBCG dataset). It shows that, on average, all the chosen models exhibited highest performance in identifying Grade 0 and lowest performance in identifying Grade 1 (except MobileNetV2).

**Figure 9:**
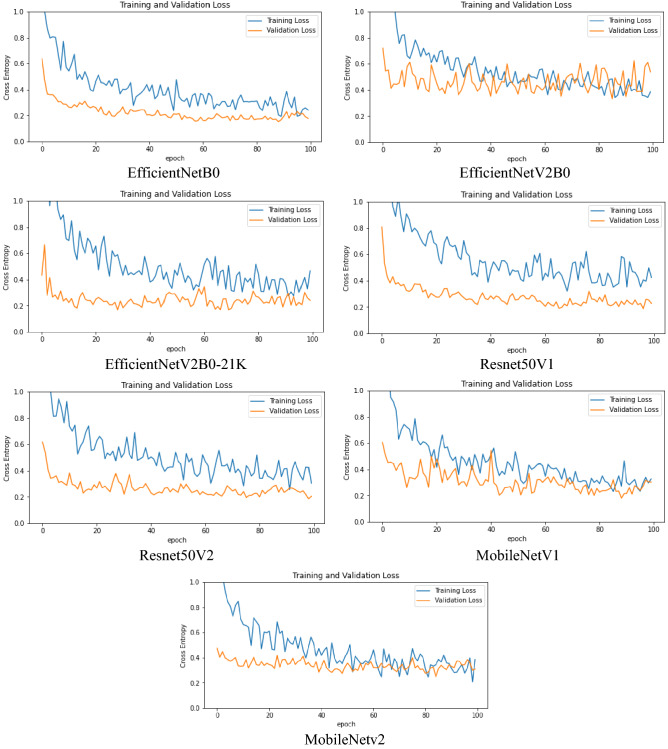
The training versus validation loss curves of the test set. Training and validation loss curves on the test set (20 percent of the FBCG dataset) for each of the seven selected comparative CNN models (trained by training set from 80% of the FBCG dataset previously used for the fivefold stratified CV). The fluctuations and volatility (noise) depicted in the curves are most likely the result of data augmentation. In general, none of the model curves indicate model overfitting since the validation loss curves are lower than the training loss curves.

Table 8 summarises the final breast cancer grading results (receiver operating characteristics (ROC)) on the test set using a model retrained with all of the images from the training set. The receiver operating characteristic (ROC) curve, shown in Fig. 8, is generated by computing and plotting the true positive rate versus the false positive rate for a binary classifier over a range of threshold values. The area under the curve (AUC) is depicted in the figure, which shows that all models perform nearly equally well, in which the Grade 0 versus other grades achieved the highest average AUC. Figure 9 depicts the training versus validation loss curve for the test set, showing the models can be built without obvious signs of being overfitted.

### Limitation of study

The dataset used in this study was inspired by Abdelli et al.^26^. As a result, the generated results are only applicable to the FBCG dataset. Additionally, the results are comparable only to Abdelli et al.^26^ using the same dataset. This research examined seven well-known and state-of-the-art CNN architectures (EfficientNetB0, EfficientNetV2B0-21k, ResNetV1-50, ResNetV2-50, MobileNetV1, and MobileNetV2); additional CNN architectures were omitted due to time constraints and limited resources. The methodology involved end-to-end feature extraction via transfer learning using pre-trained CNN architectures. However, we omitted from our work the fine-tuning of CNN architecture. If fine-tuning is performed in the correct location within the model architecture, it can improve the performance of CNNs without inducing overfitting. In our study, we omitted the effect of stain normalisation. Veta et al.^69^ asserted that the tissue preparation and histology staining processes could introduce colour discrepancies into images, impairing CNN training. However, as demonstrated in the study by Gupta et al.^70^, useful features and classifiers may obviate the need for stain normalisation.

### Challenges

One of the difficulties we encountered in this work was the issue of overfitting. The adopted dataset (FBCG dataset) is relatively small in comparison to other histopathological breast cancer datasets (BreaKHis). As a result, when training with more complex CNN architectures, overfitting may occur. To overcome this obstacle, we augmented the adopted dataset with augmentation layers (random flip, random rotation, and random zoom). Additionally, we included two dropout layers that can randomly zeros out input units at a specified rate during model training. Dealing with an unbalanced dataset is another of the difficulties encountered in this work. As a result, the CNN model is prone to predict the majority class. Thus, we applied the class weighting technique by giving the minority class a higher weight in the model cost function in order to impose a greater penalty on the minority class.

## Conclusion

In this paper, we compared the performance of seven CNN architectures in the automated IDC grading application. The Four-Breast-Cancer-Grades (FBCG) dataset was classified into four grades using transfer learning: Grade 0, Grade 1, Grade 2, and Grade 3. The results showed that EfficientNetV2B0-21k outperformed all other CNN models in the fivefold stratified CV (balanced accuracy score = 0.9666 ± 0.0185, macro precision = 0.9646 ± 0.0174, recall = 0.9666 ± 0.0185, and F1 score = 0.9642 ± 0.0184), despite having low FLOPs (0.72B), parameters (7.1 M), inference time (0.0758 ± 0.0001 s), and training time (0.5592 ± 0.0162 h). The EfficientNetV2B0-21k also achieved the highest balance accuracy (0.9524) and macro recall (0.9524) in the test. Similarly, the MobileNetV1 scored the highest balanced accuracy (0.9524), macro precision (0.9545), and macro recall (0.9545) in the test results (0.9524). All CNN models, however, demonstrated significant capability in the automated IDC grading application, with an average balanced accuracy of 0.9361 ± 0.0189 in the fivefold stratified CV and 0.9308 ± 0.0211 in the test result. Choosing heavy-weight CNNs is not a problem because the IDC grading application highlights that accuracy and resources are not limiting factors. If future IDC grading applications require real-time settings, a smaller and faster CNN (MobileNetV2) would be preferable. We may expand our work for future development by comparing it to more recent state-of-the-art CNN architectures. In addition, to conduct our comparative performance analysis, we may consider a variety of breast cancer histopathological datasets.

## Data Availability

The origin datasets combined for the current study are available in the Four Breast Cancer Grades (FBCG) Dataset https://web.inf.ufpr.br/vri/databases/breast-cancer-histopathological-database-breakhis/, and breast carcinoma histological images from the Department of Pathology, https://zenodo.org/record/834910#.WXhxt4jrPcs.
